# Screening for Resistant Germplasms and Quantitative Trait Locus Mapping of Resistance to Tomato Chlorosis Virus

**DOI:** 10.3390/ijms26052060

**Published:** 2025-02-26

**Authors:** Wenzheng Gao, Zhirong Wang, Chenchen Dong, Kai Wei, Yifan Chen, Zhuoyao Qiu, Ziteng Liu, Xin Li, Lei Liu, Yongchen Du, Zejun Huang, Junming Li, Xiaoxuan Wang

**Affiliations:** 1 State Key Laboratory of Vegetable Biobreeding, Institute of Vegetables and Flowers, Chinese Academy of Agricultural Sciences, Beijing 100081, China; gaowenzheng1996@163.com (W.G.); wangzhirong@caas.cn (Z.W.); dongchenchen0216@163.com (C.D.); 13126770229@163.com (K.W.); 2019204030@njau.edu.cn (Y.C.); 18838919798@163.com (Z.Q.); 82101225064@caas.com (Z.L.); lixin10@caas.cn (X.L.); liulei02@caas.cn (L.L.); duyongchen@caas.cn (Y.D.); huangzejun@caas.cn (Z.H.); lijunming@caas.cn (J.L.); 2 Institute of Crop Science, Chinese Academy of Agricultural Sciences, Beijing 100081, China

**Keywords:** tomato chlorosis virus, germplasm, resistance identification, RNA-seq, QTL

## Abstract

Tomato chlorosis virus (ToCV) is an emerging plant virus that poses a substantial threat to the cultivation of economically vital vegetable crops, particularly tomato (*Solanum lycopersicum*). Despite its substantial impact on crop yield, resistant or tolerant tomato germplasms have not been well documented, and the genetic basis of resistance to ToCV remains poorly understood. In this study, two wild accessions that were immune to ToCV and five accessions that were highly resistant to ToCV were identified from 58 tomato accessions. Additionally, a novel method was developed for evaluating resistance to ToCV in tomatoes, and it was observed that tomatoes exhibited typical pathological features on days 15 and 30 after ToCV inoculation, referred to as Stage 1 and Stage 2, respectively. Using quantitative trait locus (QTL) sequencing in conjunction with classical QTL approaches, ToCV resistance loci were identified in two F2 populations derived from the crosses between SG11 (susceptible) and LA1028 (resistant) and between SP15 (susceptible) and LA0444 (resistant). Genetic analysis indicated that resistance to ToCV in the wild-type ToCV-resistant tomato accessions LA1028 and LA0444 was quantitative and mainly governed by four loci (*Qtc1.1* and *Qtc11.1* from LA1028 and *Qtc7.1* and *Qtc9.1* from LA0444). Subsequently, transcriptome analysis of three resistant accessions (LA2157, LA0444, and LA1028) and two susceptible accessions (SG11 and SP15) revealed unique differentially expressed genes and specific biological processes in the two stages of ToCV infection. This study provides new resistant germplasms and potential genetic resources for ToCV resistance, which can be valuable in tomato molecular breeding programs in obtaining resistant varieties.

## 1. Introduction

Tomato yellowing virus (ToCV) belongs to the Closteroviridae family, Crinivirus genus [1,2]. The genome of ToCV is composed of two positive-sense RNAs [3], with RNA1 encoding six replication-associated proteins, and this strand containing four open reading frames (ORFs) [3], and RNA2 encoding proteins related to the movement, encapsidation, and whitefly transmission of ToCV, and this strand contains nine ORFs [4,5,6,7,8]. NbMPK3/6 directly phosphorylates ToCV P7 via the decrease in callose deposition occurring at plasmodesmata to modulate antiviral defense mechanisms, thereby facilitating the transmission of ToCV in *N. benthamiana* [9]. ToCV has spread worldwide, affecting regions including North America, Asia, and Europe [2,10,11,12,13,14].

ToCV causes interveinal leaf chlorosis, leaf bronzing, and chlorotic flecking, all of which can lead to reduced photosynthesis in tomatoes [15]. These symptoms mainly appear on the old leaves of tomato plants, with the upper parts of the plants showing milder symptoms. The symptoms of ToCV are similar to those of nutrient deficiency symptoms and some physiological diseases, which may lead to early senescence and yield reduction in tomatoes [16]. Infection with ToCV can also reduce the yield of peppers, indicating that the harm caused by ToCV is more widespread [17]. ToCV significantly affects the production of vegetables, which requires attention and the search for solutions [18].

ToCV has the ability to be transmitted in a semi-persistent manner by *Bemisia tabaci* biotypes A, B [19], and Q [13], as well as *Trialeurodes abutilonea* and *Trialeurodes vaporariorum* [19]. Suppressing the population of whiteflies through physical and chemical control is one method to control ToCV [20]. However, physical and chemical control methods, while capable of suppressing the population of whiteflies and playing a certain role in controlling ToCV in vegetable production, still have unsatisfactory control effects. Even a small number of surviving whiteflies carrying ToCV can effectively transmit it [21]. Screening for ToCV-resistant germplasms and mapping resistance QTLs are effective means for controlling ToCV, but there is limited research on ToCV resistance. Two sources of ToCV resistance were pinpointed by García-Cano et al. through the screening of resistant germplasms: one from the *Solanum peruvianum* LA0444 and the other from the *Solanum chmielewskii* LA1028 [22]. The research by Mansilla-Cordova et al. also verifies this finding [23]. Furthermore, studies have shown that SlSKIP2 knockout exhibits better plant phenotypes and higher chlorophyll contents compared to controls after infection with ToCV [24].

Up until now, there were no commercial tomato cultivars resistant to ToCV. Screening for ToCV-resistant germplasms and mapping ToCV resistance QTL are necessary for breeding ToCV-resistant cultivars. In this study, we screen ToCV-resistant germplasms and conduct QTL mapping for ToCV resistance. Utilizing the QTL-seq method and map-based cloning, we mapped four ToCV-resistant QTLs, which are *QTC11.1* and *QTC1.1* derived from LA1028, and *QTC9.1* and *QTC7.1* derived from LA0444. Transcriptome analysis based on RNA sequencing (RNA-seq) is used to explore the pathogenesis of ToCV, as well as the anti-ToCV mechanisms of LA1028 and LA0444.

## 2. Results

### 2.1. Evaluation of Germplasm Resources Resistant to ToCV in Tomato

A total of 58 germplasms were used for ToCV resistance resource screening, including 21 cultivated tomatoes, 15 *S. peruvianum*, 1 *S. corneliomulleri*, 8 *S. pimpinellifolium*, 2 *S. chilense*, 8 *S. habrochaites*, 1 *S. cheesmanii*, 1 *S. chmielewskii*, and 1 *S. pennellii* (Table 1). The screening revealed two immune genotypes (*S. peruvianum* LA2157 and LA0444) demonstrating complete immunity (DSI = 0), along with five highly resistant accessions (*S. chmielewskii* LA1028 and *S. peruvianum* LA1283, LA3858, 162-3, and Wo587), offering valuable genetic germplasms for resistance gene identification. Eleven accessions—namely two *S. habrochaites* 162-40 and LA1777; two *S. peruvianum* Wo596 and Wo034; two *S. chilense* LA1932 and LA1969; *S. cheesmanii* LA0317; *S. pennellii* LA0716; and cultivated lines LA0490, CLN3024F2-104-48-1-0, and 031-D62—exhibited slight chlorotic spots on leaves 50 days after inoculation. Sixteen accessions—namely cultivated lines LA2827, LA2830, LA2821, and LA2830; two *S. pimpinellifolium* LA1589-3 and LA0722; six S. peruvianum Wo015, Wo589, Wo606, Wo607, Wo677, and Wo688; and four *S. habrochaites* 162-38, 162-39, 162-41, and 162-42—exhibited dark green and yellowing veins on lower leaves 50 days after inoculation. Thirteen accessions—namely cultivated lines LA0154, Fla.MH-1, LA2823, and Zaofen2; five *S. pimpinellifolium* 162-5, LA1245, LA1246, LA2093, and LA2097; two *S. peruvianum* Wo030, SP15; two *S. habrochaites* LA1033 and 162-37—were susceptible to ToCV. Eleven cultivated accessions showed high susceptibility to ToCV. In the late development stage, they exhibited yellowing and chlorosis on the leaves of the whole plant, which had a significant impact on photosynthesis during this period.

Statistical validation among different ToCV resistance phenotypes indicates that there is no significant difference between immune (I) and highly resistant (HR) phenotypes, while there are significant differences between I and HR and other phenotype groups (R, resistance; MR, moderate resistance; S, susceptible; HS, highly susceptible) (*p* < 0.0001), which suggests that germplasms with phenotypes I and HR can be applied in the mapping of ToCV-resistant gene QTLs and the breeding of ToCV-resistant varieties (Figure S2).

### 2.2. Establishing a ToCV Inoculation and Evaluation Method

The natural inoculation of ToCV exhibits significant environmental variability and unpredictability, making it difficult to meet the research conditions for the pathogenesis or resistance mechanisms of ToCV. We established a new method for ToCV inoculation and evaluation in the hope of addressing the aforementioned issues. Experimental seedlings in the three-leaf developmental stage received controlled ToCV exposure through cyclical releases of ToCV-infected Bemisia tabaci vectors over a 30-day inoculation protocol. At the conclusion of the inoculation access period (IAP), each plant released approximately 400 viruliferous whiteflies. Subsequent to the IAP, imidacloprid was applied to the plants, and then they were rinsed with water to ensure that the phenotype was not affected by the imidacloprid, and symptom development and virus detection were monitored.

**Figure 1:**
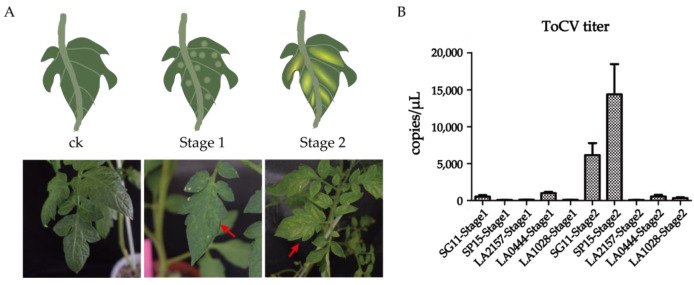
ToCV inoculation and evaluation were carried out on SG11, SP15, LA2157, LA0444, and LA1028. (**A**) On the left are healthy tomato leaves. In the middle are leaves in the chlorotic spot stage, and on the right are leaves in the chlorosis stage. The red arrow points to the symptoms of ToCV. (**B**) Absolute quantification of ToCV in SG11, SP15, LA2157, LA0444, and LA1028 in stages 1 and 2.

After inoculating ToCV on two susceptible accessions (S11 and SP15) and three resistant accessions (LA0444, LA1028, LA2157), the symptoms were continuously assessed. The results indicated that the disease progression of ToCV can be divided into two key stages. Tomato leaves of susceptible accessions SP15 and SG11 exhibit chlorotic spots 15 days after inoculation with ToCV, a stage with typical symptoms that we refer to as the ToCV chlorotic spot stage (Stage 1) (Figure 1A and Figure S3). After 30 days of inoculation with ToCV, the number of chlorosis spots on the leaves began to significantly increase, merging and deepening in color, manifested as yellowing between the leaf veins. This characteristic stage with typical symptoms is called the ToCV chlorosis stage (Stage 2) (Figure 1A and Figure S3). ToCV-resistant accessions LA2157, LA0444, and LA1028 displayed no typical symptoms in Stage 1 and Stage 2 (Figure S3). The typical symptoms of these two stages can be used for the early diagnosis of ToCV and to assess the time and severity of the infection.

To achieve the absolute quantification of ToCV titers, RT-qPCR was carried out. Seven concentration gradients were set up with the range of 5.6 × 10^3^ to 5.6 × 10^9^ copies/μL. The standard curve exhibited a uniform tendency in which the crossing point (CP) value rose as the concentration of the template declined. This indicated a linear association between the CP value and the titer of ToCV (Figure S4). The results of ToCV absolute quantification indicate that in the susceptible accessions SP15 and SG11, the titer of ToCV in Stage 2 was significantly higher than in Stage 1. And the resistant accessions LA1028, LA0444, and LA2157 maintained a low titer in Stage 1 and Stage 2 (Figure 1B). These findings indicate that RT-qPCR is a high-sensitivity method for ToCV detection. Furthermore, the resistant accessions exhibited low ToCV titers in both stages, suggesting that the resistant accessions can maintain a lower viral level. Our data suggest that maintaining low virus titers is associated with ToCV resistance, but further research is needed to confirm causal mechanisms.

### 2.3. Mapping of QTLs Associated with ToCV Resistances

**Figure 2:**
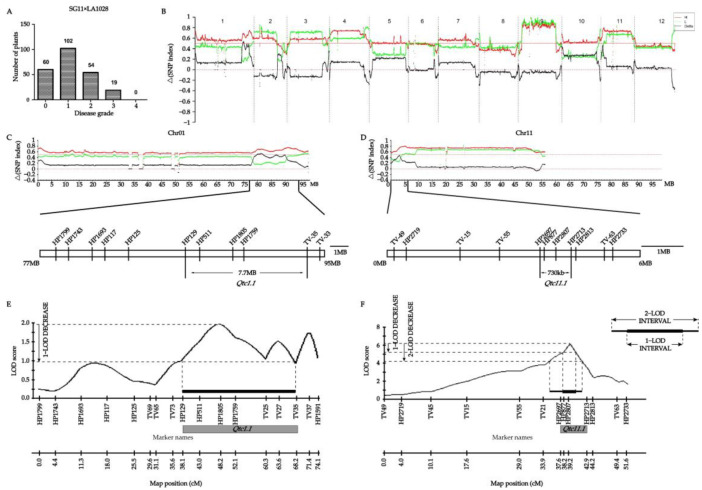
Mapping of *Qtc1.1* and *Qtc11.1*. (**A**) Histograms depicting the distribution of ToCV disease grades within the SG1 × LA1028 populations. (**B**) In the QTL-seq diagrams, the positions on the 12 chromosomes are indicated by the X-axis, and the Y-axis represents Δ(SNP index). The black line shows Δ(SNP index), the red line shows the SNP index of the resistance pool, while the green line shows the SNP index of the susceptibility pool. (**C**) In the QTL-seq diagrams related to *Qtc1.1*, the position (Mb) on chromosome 1 is denoted by the X-axis, while the Y-axis represents the Δ(SNP index). Based on these diagrams, *Qtc1.1* was mapped to an approximately 7.3 Mb region flanked by HP129 and TV35. (**D**) In the QTL-seq diagrams for *Qtc11.1*, the X-axis denotes the position (Mb) on chromosome 11, and the Y-axis represents the Δ(SNP index). Based on these diagrams, *Qtc11.1* was mapped to an approximately 730 kb region flanked by HP2697 and HP2713. (**E**,**F**) Linkage analysis of the locations of *Qtc1.1* (**E**) and *Qtc11.1* (**F**) by molecular markers. Note: 1-LOD: 97% confidence interval; 2-LOD: 99.8% confidence interval.

For the purpose of mapping the ToCV resistance genes derived from LA1028 (with a DSI of 9.4), an F2 population was created by crossing it with the highly susceptible accession SG11 (which had a DSI of 90.6) (Figure S5A). The frequency distributions exhibited continuous variation for the character (Figure 2A), suggesting that the ToCV resistance derived from LA1028 was a quantitatively inherited trait. After that, whole-genome sequencing was carried out on two extreme trait pools, a resistant pool and a susceptible pool, which were composed of the F2 individuals with extreme phenotypes. QTL-seq analysis demonstrated that in a 15 Mb chromosome segment on chromosome 1, spanning from 77 to 92 Mb, and a 3 Mb segment on chromosome 11, ranging from 0 to 6 Mb, the ΔSNP index values surpassed 0.5. Additionally, in an 11 Mb chromosome segment on chromosome 2, between 38 and 49 Mb, the ΔSNP index values approached 0.4. Similarly, in a 3 Mb chromosome segment on chromosome 10, from 0 to 3 Mb, and a 5 Mb chromosome segment on chromosome 12, between 62 and 67 Mb, the ΔSNP index values were approximately 0.4 and −0.4, respectively (Figure 2B–D).

InDel markers were used in genetic analysis to validate QTLs detected by QTL-seq. The QTL located on chromosome 1 was mapped to a 7.3 Mb region situated between markers HP129 and TV35. The LOD score of this was 1.96, and it accounted for 3.8% of the phenotypic variance (Figure 2C,E). This QTL was named *Qtc1.1*. The major QTL on chromosome 11 was mapped to a 730 kb region situated between markers HP2697 and HP2713. The LOD score of this was 6.05, and it accounted for 11.4% of the phenotypic variance (Figure 2D,F). This QTL was named *Qtc11.1*. The findings of the genetic analysis indicated that the LOD scores of the three loci on chromosomes 2, 10, and 12 were approximately 1.0, suggesting a weak correlation with tomato disease resistance. In future research, we will concentrate on these QTL loci.

These findings indicate that *Qtc1.1* as well as *Qtc11.1* from LA1028 are significant.

**Figure 3:**
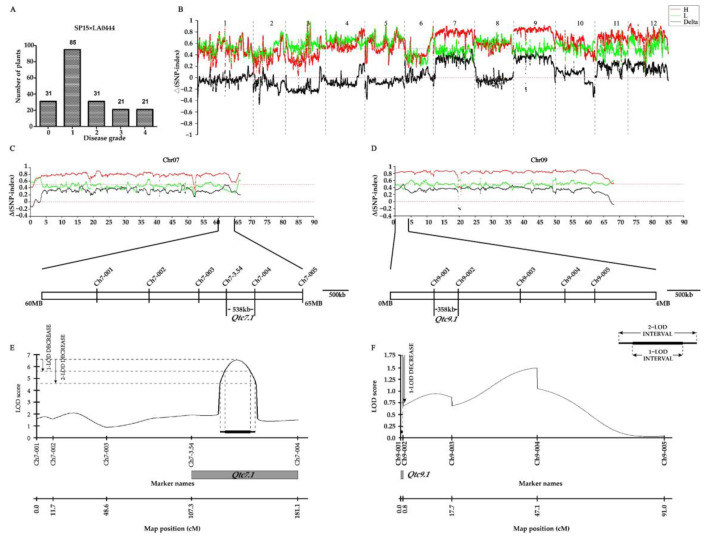
Mapping of *Qtc7.1* and *Qtc9.1*. (**A**) Histograms showing the distribution of ToCV disease grades within the SP15 × LA0444 populations. (**B**) In the QTL-seq diagrams, the positions on the 12 chromosomes are indicated by the X-axis, and the Y-axis represents Δ(SNP index). The black line shows Δ(SNP index), the red line shows the SNP index of the resistance pool, while the green line shows the SNP index of the susceptibility pool. (**C**) In the QTL-seq diagrams related to *Qtc7.1*, the position (Mb) on chromosome 7 is denoted by the X-axis, while the Y-axis represents Δ(SNP index). Based on these diagrams, *Qtc7.1* was mapped to an approximately 538 kb region flanked by Ch7-3.54 and Ch7-004. (**D**) In the QTL-seq diagrams related to *Qtc9.1*, the position (Mb) on chromosome 9 is denoted by the X-axis, while the Y-axis represents Δ(SNP index). Based on these diagrams, *Qtc9.1* was mapped to an approximately 358 kb region flanked by Ch9-001 and Ch9-002. (**E**,**F**) Linkage analysis of the locations of *Qtc7.1* (**E**) and *Qtc9.1* (**F**) by molecular markers. Note: 1-LOD: 97% confidence interval; 2-LOD: 99.8% confidence interval.

For the purpose of mapping the ToCV resistance genes derived from LA0444 (with a DSI of 0), an F2 population was created by crossing it with the highly susceptible accession SP15 (which had a DSI of 80) (Figure S5B). The frequency distributions exhibited continuous variation for the character (Figure 3A), suggesting that the ToCV resistance inherited from LA0444 was a quantitatively inherited trait. One was a resistant pool, and the other was a susceptible pool, which were composed of the F2 individuals with extreme phenotypes. QTL-seq analysis indicated that the Δ(SNP index) values were greater than 0.5 in roughly 5 Mb intervals from 60 to 65 Mb on chromosome 7 and from 0 to 5 Mb on chromosome 9 (Figure 3B–D). To verify the two QTLs detected via QTL-seq, InDel markers were employed for genetic analysis. The QTL on chromosome 7 was mapped within a 538 kb region that was located between the markers Ch7-3.54 and Ch7-004. It had an LOD score of 6.52 and accounted for 5.4% of the phenotypic variance. This QTL was named *Qtc7.1* (Figure 3C). The QTL on chromosome 9 was mapped within a 358 kb region that was located between the markers Ch9-001 and Ch9-002. It had an LOD score of 1.75 and accounted for 4.45% of the phenotypic variance. This QTL was named *Qtc9.1* (Figure 3D). These results suggest that *Qtc7.1* as well as *Qtc9.1* originating from LA0444 are significant.

### 2.4. Analysis of Candidate QTLs for ToCV Resistance Based on Expression Profiles

**Figure 4:**
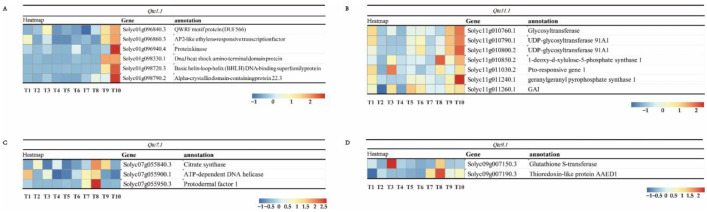
A heatmap was generated to display the expression of DEGs within the ToCV QTLs across different samples: SG11 in Stage 1 and Stage 2, SP15 in Stage 1 and Stage 2, LA2157 in Stage 1 and Stage 2, LA0444 at Stage 1 and Stage 2, and LA1028 in Stage 1 and Stage 2. Additionally, annotations of these DEGs were provided. (**A**) A heatmap was created to show the expression of DEGs in *Qtc1.1*, and the DEGs were also annotated. (**B**) A heatmap depicting the expression of DEGs within *Qtc11.1* was generated, along with the annotation of these DEGs. (**C**) A heatmap depicting the expression of DEGs within *Qtc7.1* was generated, along with the annotation of these DEGs. (**D**) A heatmap depicting the expression of DEGs within *Qtc9.1* was generated, along with the annotation of these DEGs. Note: T1 (SG11.Stage 1), T2 (SG11.Stage 2), T3 (SP15.Stage 1), T4 (SP15.Stage 2), T5 (LA2157.Stage 1), T6 (LA2157.Stage3), T7 (LA0444.Stage 1), T8 (LA0444.Stage 2), T9 (LA1028.Stage 1), and T10 (LA1028.Stage 2).

The methods of QTL-seq and QTL mapping were applied to analyze the F2 populations in order to identify ToCV-resistant QTLs. For the purpose of identifying resistance genes, expression profiling analysis was carried out on the genes within the four candidate QTLs, with the aim of assisting in the mapping of ToCV-resistant QTLs and predicting candidate genes within these QTLs. In *Qtc1.1*, a total of 373 genes were annotated, and six of them showed distinct expression patterns (Table S6). During stages 1 and 2, the expression levels of six genes (*Solyc01g096840*, *Solyc01g096860*, *Solyc01g096940*, *Solyc01g098330*, *Solyc01g098720*, and *Solyc01g098790*) were greater in LA1028 as opposed to those in SP15 and SG11 (Figure 4A). In *Qtc11.1*, a total of 77 genes were annotated. Among them, *Solyc11g010760*, *Solyc11g010790*, *Solyc11g010800*, *Solyc11g010850*, *Solyc11g011240*, and *Solyc11g011260* exhibited greater expression levels in LA1028 compared to SP15 and SG11 in Stage 1 and Stage 2 (Figure 4B, Table S7). Additionally, *Solyc11g011030* had a higher expression in LA1028 compared to SG11 and SP15 during Stage 2 (Figure 4B).

In *Qtc7.1*, a total of 65 genes were annotated. Among them, Solyc07g055840 demonstrated greater expression levels in LA0444 during Stage 2 as compared to SP15 and SG11 (Figure 4C, Table S8). Moreover, *Solyc07g055900* had a higher expression in LA0444 than in SP15 and SG11 throughout Stage 1 and Stage 2 (Figure 4C).

Among these genes, *Solyc01g096940* encodes a protein kinase and is a membrane-bound protein. This is a common characteristic feature among numerous disease-resistant genes. *Solyc11g011030* is capable of regulating the defense response and the jasmonic acid-mediated signaling pathway. *Solyc07g055950* is protodermal factor 1, which might be associated with the generation of epidermal cells [25]. *Solyc09g007190* is a thioredoxin-like protein that is situated in chloroplasts. It may have an impact on photosynthetic efficiency (Figure S6). These findings can play a key role in optimizing the mapping of ToCV resistance genes.

### 2.5. Transcriptome Analysis of Multiple Accessions Infected with ToCV

In order to investigate the pathogenesis and resistance mechanism of ToCV, resistant accessions LA2157, LA0444, and LA1028, as well as susceptible accessions SP15 and SG11, were used as experimental materials. The non-infected group (inoculated with non-viruliferous whiteflies) was used as a control. Samples were taken in the first and second stages after ToCV infection, and RNA sequencing analysis was performed on them.

**Figure 5:**
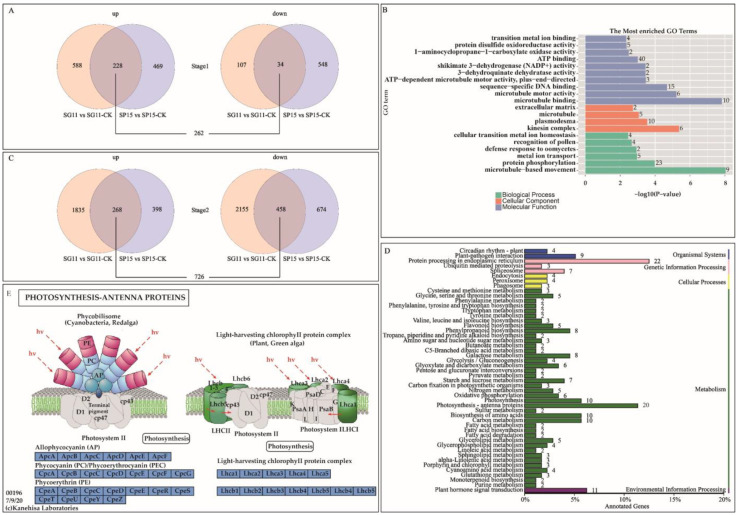
Venn diagrams were used to display the DEGs, and functional annotation of the genes was also carried out. (**A**) A Venn diagram is presented to illustrate the genes with upregulated expression on the left and downregulated expression on the right. These genes are compared between the experimental and control groups of SP15 and SG11 in Stage 1 (where CK represents the control groups). (**B**) A GO enrichment map was constructed to analyze the common DEGs between SP15 and SG11 in Stage 1. (**C**) A Venn diagram is presented to illustrate the genes with upregulated expression on the left and downregulated expression on the right. These genes are compared between the experimental and control groups of SP15 and SG11 in Stage 2 (where CK represents the control groups). (**D**) A KEGG enrichment map was generated to analyze the common differentially expressed genes (DEGs) between SP15 and SG11 in Stage 2. (**E**) A diagram depicting the photosynthesis antenna protein pathway (ko00196) is presented, and the positions of various genes within this pathway are indicated in the blue box. Note: In “The Most Enriched GO Terms”, blue terms represent molecular function, orange terms represent cellular component, and green terms represent biological process.

During Stage 1, a total of 262 DEGs were detected in the overlapping region of the infected SP15 and SG11 groups and were compared with their corresponding control groups (Figure 5A). The GO annotation results revealed that numerous DEGs are related to microtubule-based movement, microtubule binding, and microtubule motor activity. This indicates that in the susceptible accessions SG11 and SP15, ToCV infection upregulates gene expression associated with the microtubule system (Figure 5B). During Stage 2, a total of 726 DEGs were found in the overlapping region of SP15 and SG11 when these were compared with their respective control groups (Figure 5C). In ToCV-infected tomatoes during this stage, the expression levels of genes encoding photosynthesis-antenna proteins were notably reduced (Figure 5E). KEGG pathway analysis showed that the DEGs were enriched in pathways associated with plant–pathogen interactions, photosynthesis-antenna proteins, brassinosteroid biosynthesis, and plant hormone signal transduction (Figure 5D,E).

These results imply that during Stage 1, ToCV infection might cause the upregulation of genes related to the microtubule system. Certain plant viruses employ the microtubule system to move from cell to cell [26,27]. In Stage 2, ToCV infection has a severe impact on the photosynthetic system of tomatoes, resulting in a decline in the efficiency of photosynthesis. Hence, the reduced expression levels of genes associated with the photosynthesis-antenna protein may be a factor contributing to chlorosis, which requires further study.

**Figure 6:**
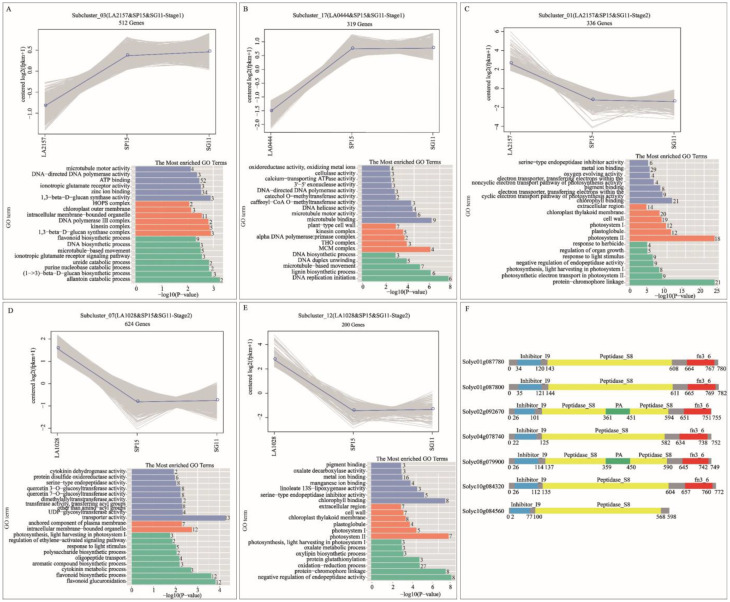
Analysis of the co-expression trends of DEGs and GO annotation were carried out. (**A**) In Stage 1, a trend diagram for subcluster_03 (comprising LA2157, SP15, and SG11) was obtained from the co-expression trend analysis. Additionally, GO enrichment analysis was conducted. (**B**) In Stage 1, a trend diagram for subcluster_17 (LA0444, SP15, and SG11) was obtained from the co-expression trend analysis. Additionally, GO enrichment analysis was conducted. (**C**) In Stage 2, a trend diagram for subcluster_01 (LA2157, SP15, and SG11) was obtained from the co-expression trend analysis. Additionally, GO enrichment analysis was conducted. (**D**) In Stage 2, a trend diagram for subcluster_07 (LA1028, SP15, and SG11) was obtained from the co-expression trend analysis. Additionally, GO enrichment analysis was conducted. (**E**) In Stage 2, a trend diagram for subcluster_12 (LA1028, SP15, and SG11) was obtained from the co-expression trend analysis. Additionally, GO enrichment analysis was conducted. (**F**) Pfam annotation of *Solyc01g08780.2*, *Solyc01g087800.2*, *Solyc02g092670.1*, *Solyc04g078740.2*, *Solyc08g079900.3*, *Solyc10g084320.3*, and *Solyc10g084560.3.* Note: In “The Most Enriched GO Terms”, blue terms represent molecular function, orange terms represent cellular component, and green terms represent biological process.

In Stage 1, when LA2157 was compared with SP15 and SG11, 2996 and 2629 differentially expressed genes (DEGs) were identified, respectively. These genes were then classified into 16 expression clusters (Figure S7). Specifically, in subcluster_03, there were 512 genes that showed lower expression levels in LA2157 than in SP15 and SG11 during Stage 1. These genes were found to be enriched in microtubule-based movement and microtubule motor activity (Figure 6A). Moreover, in Stage 1, when LA0444 was compared with SP15 and SG11, 4104 and 3561 differentially expressed genes (DEGs) were detected, respectively. These genes were then categorized into 19 expression clusters (Figure S8). Specifically, in subcluster_17, 319 genes exhibited lower expression levels in LA0444 than in SP15 and SG11 during Stage 1. These genes were enriched in microtubule-related activities (Figure 6B). In Stage 2, when LA2157 was compared with SP15 and SG11, 6260 and 2909 differentially expressed genes (DEGs) were identified, respectively. Subsequently, these genes were classified into 12 expression clusters (Figure S9). Specifically, in subcluster_01, 336 genes had higher expression levels in LA2157 than in SP15 and SG11 during Stage 2. These genes were enriched in the negative regulation of endopeptidase activity and serine-type endopeptidase inhibitor activity (Figure 6C). In Stage 2, when LA1028 was compared with SP15 and SG11, 6076 and 4548 differentially expressed genes (DEGs) were identified, respectively. These genes were then divided into 15 expression clusters (Figure S10). In Stage 2, subclusters 07, 08, and 12 showed distinct expression patterns when comparing SP15, SG11, and LA1028 (Figure S10). During this stage, in subcluster 12, 200 genes had higher expression levels in LA1028 compared to those in SP15 and SG11 (Figure 6E). The GO annotation results revealed that the genes within subcluster 12 were mainly associated with the negative regulation of endopeptidase activity (Figure 6E). In subcluster 07, a total of 624 genes showed higher expression levels in LA1028 than in SP15 and SG11 (Figure 6D). Within subcluster 07, eight genes (*Solyc01g087780*, *Solyc01g087800*, *Solyc02g092670*, *Solyc04g078740*, *Solyc08g079900*, *Solyc10g084560*, and *Solyc10g084320*) carried the peptidase inhibitor I9 domain (Inhibitor_I9) and were related to serine-type endopeptidase activity (Figure 6D,F).

These findings suggested that during Stage 2, the genes associated with peptidase inhibitors showed lower expression levels in LA2157 and LA1028 compared to the susceptible accessions. This suggests that the expression level of genes related to peptidase inhibition could be a contributing factor to ToCV resistance.

As shown in Figure S11, the RT-qPCR results indicate that the transcript levels of the selected genes were consistent with the RNA-seq data, which demonstrated the dependability of the RNA-seq results (the primer information for RT-qPCR is given in Table S9). These results offer reference data and an expression profile for the further investigation of the mechanisms of ToCV resistance, facilitating the identification of resistance genes.

## 3. Discussion

ToCV significantly affects vegetable production, making research on ToCV very meaningful [11]. Screening resistant germplasms will contribute to the mapping of ToCV resistance QTLs, the creation of breeding materials resistant to ToCV, and the study of the mechanisms of ToCV resistance. Our research findings are consistent with previous studies, with LA1028 and LA0444 showing high resistance to ToCV. Furthermore, we have also identified four accessions (LA1283, LA3858, Wo587, and 162-3) that exhibit high resistance to ToCV, and LA2157 shows immunity to ToCV.

The ToCV infection has a long incubation period, and early infection with ToCV may be overlooked, thereby missing the best time for ToCV prevention and control. It is necessary to establish ToCV inoculation, evaluation, and detection methods, which can identify the ToCV infection in the early stages, and timely control measures can be taken. In this study, we developed a ToCV inoculation and evaluation method, which can help us explore the pathological characteristics of ToCV and the resistance mechanism of ToCV-resistant materials.

In this study, we conducted ToCV inoculation and evaluation in an insect-proof glasshouse to ensure the reliability of screening resistant germplasms and the QTLs mapping of resistance to ToCV. However, inoculating ToCV in an insect-proof glasshouse cannot unify the onset time of ToCV, and thus detailed research work still cannot be completed. This is because the study of the resistance and pathogenesis mechanisms of ToCV requires a relatively consistent onset time. Therefore, we established a ToCV inoculation and evaluation method. For virus inoculation, healthy whitefly adults were fed on ToCV-infected plants in insect-proof cages for 48 h to ensure that the whiteflies carried ToCV. Over the course of 30 days, the test plants endured continuous inoculation procedures. At three-day intervals, viruliferous whiteflies were introduced. By the conclusion of the IAP, each plant had been exposed to around 400 viruliferous whiteflies in total. This inoculation method can ensure a high inoculation rate for ToCV, maintain a consistent inoculation time, and minimize the impact of environmental factors. At the same time, this inoculation method can, to some extent, reduce the impact of the feeding choice of whiteflies on ToCV evaluation. During the ToCV disease period, we can observe the typical characteristics of tomato infected with ToCV in various stages of onset and conduct pathological studies on the stages of interest. In accordance with the symptoms noticed in ToCV-infected plants, the disease progression was classified into two essential stages, namely the ToCV chlorotic spot stage (Stage 1) after 15 days of inoculation and the ToCV chlorosis stage (Stage 2) after 30 days of inoculation. The ToCV-resistant accessions LA0444, LA2157, and LA1028 exhibited no symptoms in Stage 1 and Stage 2.

RT-qPCR was used to quantify the titer of ToCV. In susceptible tomato, the titer of ToCV during Stage 1 was lower than that during Stage 2. Furthermore, the ToCV-resistant materials LA0444, LA1028, and LA2157 had lower viral titers than the susceptible materials during both stages. Our data suggest that maintaining low virus titers is associated with ToCV resistance, but further research is required to confirm causal mechanisms.

The control of ToCV is very important in tomato production, and the mapping of ToCV-resistant QTL remains the most promising method for ToCV control. In this study, we mapped ToCV-resistant QTLs from LA1028 and LA0444. In recent years, the co-infection of tomatoes by Tomato yellow leaf curl virus (TYLCV) and ToCV has become increasingly prevalent, and thus symptoms are worsening [28]. In the present study, we noticed that the simultaneous infection of tomatoes by ToCV and TYLCV could have an impact on the veracity of evaluating ToCV-resistant germplasms. Therefore, in order to reduce the impact of TYLCV infection on the identification of ToCV resistance, we selected the TYLCV-resistant accession SG11 as a parent for mapping the QTL associated with ToCV resistance. The ToCV evaluation results of the F2 population from the cross between LA1028 and SG11 follow a normal distribution, indicating that ToCV resistance derived from LA1028 is a quantitative trait controlled by multiple genes. We identified two ToCV resistance QTLs derived from LA1028, namely *Qtc1.1* on chromosome 1 and Qtc11.1 on chromosome 11. We located *Qtc1.1* in the 7.8 MB interval between HP129 and TV35. *Qtc11.1* is located in the 730 KB interval between HP2697 and HP2713. The LOD score of *Qtc11.1* is 6.05, explaining 11.4% of the phenotypic variance, indicating that *Qtc11.1* may be a major-effect QTL for ToCV resistance, whereas the LOD score of *Qtc1.1* is 1.96, explaining 3.8% of the phenotypic variance, suggesting that *Qtc1.1* might be a minor-effect QTL for ToCV resistance. Next, we will continue to develop molecular markers and then narrow down the mapping intervals for *Qtc1.1* and *Qtc11.1* to reduce the recombination rate between molecular markers and ToCV-resistant QTLs. We believe that introgressing *Qtc11.1* into susceptible tomatoes may confer a certain level of resistance to ToCV, while on the other hand, the mapping of *Qtc1.1* is also meaningful, as the accumulation of minor-effect QTL can also enhance ToCV resistance. Moreover, there is no phenomenon of hybrid sterility between *S. chmielewskii* and cultivated tomatoes, making it relatively easy to introgress ToCV-resistant QTLs from LA1028 into cultivated tomatoes. Introgressing *Qtc11.1* and *Qtc1.1* into cultivated tomatoes can provide them with certain ToCV resistance. We have started constructing a BC population with *Qtc11.1* and *Qtc1.1* introgression fragments using SG11 as the recurrent parent.

Due to the fertility barrier between *S. peruvianum* LA0444 and cultivated tomato, it is difficult to construct an F2 mapping population using *S. peruvianum* and cultivated tomato as parents. A susceptible *S. peruvianum* SP-15 was found through material screening, and using LA0444 and SP-15 as parents to construct a population can overcome fertility barriers (Table 1). The ToCV evaluation results of the F2 population from the cross between LA0444 and SP-15 follow a normal distribution, indicating that ToCV resistance derived from LA0444 is a quantitative trait controlled by multiple genes. We identified two ToCV-resistant QTLs derived from LA0444, namely *Qtc7.1* on chromosome 7 and *Qtc9.1* on chromosome 9. We located *Qtc7.1* in the 538 KB interval between Ch7-3.54 and Ch7-004. The LOD score of *Qtc7.1* is 6.52, indicating that there is indeed a QTL for ToCV resistance on chromosome 7. *Qtc7.1* can explain 5.4% of the phenotypic variance, which may suggest that *Qtc7.1* is a minor-effect QTL, and we found that there are some regions with high Δ(SNP index) on chromosome 7; the Δ(SNP index) values in a 5 Mb chromosome interval from 15 to 20 Mb and in a 5 Mb interval from 45 to 50 Mb exceeded 0.5, which may contain ToCV-resistant QTLs. Additionally, in the QTL-seq results of the LA1028 cross, no segments with high Δ(SNP index) values were found on chromosome 7, indicating that *Qtc7.1* originates from *S. peruvianum*, and no such QTL was identified in *S. chmielewskii*. In the next step, we will further develop the molecular markers within the *Qtc7.1* interval to narrow the mapping region, and at the same time, further explore other potential QTLs on chromosome 7. *Qtc9.1* is located in the 358 KB interval between Ch9-001 and Ch9-002. The LOD score of *Qtc9.1* is 1.75, explaining 4.45% of the phenotypic variance, indicating that there might be a minor-effect QTL on chromosome 9. However, a lower LOD score may indicate a higher recombination rate between the molecular marker and *Qtc9.1*, or the presence of ToCV-resistant QTLs at other loci on chromosome 9. To address this issue, we plan to further develop molecular markers within the 4 Mb segment between 0 and 4 Mb to reduce the recombination rate of *Qtc9.1*. Furthermore, we intend to develop molecular markers in other segments with high Δ(SNP index) values on chromosome 9, specifically the 35–40 MB and 45–50 MB regions, to explore additional potential QTLs for ToCV resistance. Additionally, in the QTL-seq results of the LA1028 cross, no segments with high Δ(SNP index) values were found on chromosome 9, indicating that *Qtc9.1* originates from *S. peruvianum*, and no such QTL was identified in *S. chmielewskii*.

Although the phenotypic variance explained by *QTC1.1* and *QTC9.1* is relatively low, being minor-effect ToCV-resistant QTLs, the mapping of these minor-effect ToCV-resistant QTLs is also meaningful. By aggregating major-effect and multiple minor-effect ToCV-resistant QTLs, better ToCV resistance can be produced.

There is a hybrid sterility between *S. peruvianum* and cultivated tomato, but F1 hybrids can be obtained through embryo rescue. Next, we will develop molecular markers within the mapping interval and perform fine mapping on the two QTLs mentioned above in order to clone the ToCV resistance gene derived from LA0444. In addition, we plan to combine embryo rescue to introgress the segment containing the ToCV resistance QTL from LA0444 into cultivated tomatoes.

Through transcriptome analysis, we explored the pathogenic mechanism of ToCV and the resistance mechanisms of *S. peruvianum* and *S. chmielewskii*. The results of the transcriptome analysis indicated that the expression levels of microtubule system-related genes were upregulated in the susceptible material during Stage 1 post-infection with ToCV, but not in Stage 2. The titer of ToCV in Stage 2 was also significantly higher than that in Stage 1. Studies have shown that plant viruses can achieve cell-to-cell movement via the microtubule system [26,27]. These findings suggest that ToCV may rapidly spread via the microtubule system during Stage 1 and experience explosive growth in Stage 2, and these hypotheses require further validation.

In Stage 2, the expression levels of photosynthesis-related genes in the susceptible material are downregulated, indicating that ToCV infection may affect the photosynthetic system in tomatoes. It has been demonstrated by studies that when the expression of photosynthesis-antenna protein genes is inhibited, it can cause the tea tree leaves to turn yellow [29]. Therefore, the leaf yellowing of tomatoes after ToCV infection may be related to the downregulated expression of genes related to the photosynthesis-antenna protein, which still requires further investigation.

Quantification of ToCV titer and transcriptome analysis were used to analyze the resistance mechanism of ToCV-resistant accessions. The results indicated that the ToCV titers of the resistant accessions LA2157 and LA0444 were significantly lower than those of the susceptible materials in Stage 2. The expression levels of microtubule system-related genes in LA0444 and LA2157 were lower than those in the susceptible materials. This suggests that ToCV in the susceptible accessions may utilize the microtubule system to achieve cell-to-cell movement, whereas in LA2157 and LA0444, the ability of ToCV to utilize the microtubule system for movement may be inhibited. These hypotheses require further research for confirmation.

Peptidases play an important role in the formation process of certain viruses, and peptidase inhibitors can inhibit the assembly of viruses to some extent. Despite the fact that peptidase inhibitors are applied in antiviral drugs, their application in plant virology has received relatively little research attention [30,31]. In Stage 2, the expression levels of peptidase inhibitor-related genes in the ToCV-resistant accessions LA1028 and LA2157 were lower than those in the susceptible accessions, indicating that peptidase inhibitor-related genes are associated with the formation of ToCV resistance, which still requires further investigation. These insights provide new perspectives for the study of ToCV resistance.

Overall, the findings of this study provide a material and theoretical basis for ToCV resistance breeding, as well as a novel method for ToCV inoculation and evaluation, which may contribute to the early control of ToCV. Additionally, transcriptome analysis provides new insights into the pathological characteristics and resistance mechanisms of ToCV.

## 4. Materials and Methods

### 4.1. Germplasm Screening

In this study, 58 genotypes were sourced from the Institute of Vegetables and Flowers, Chinese Academy of Agricultural Sciences. Furthermore, in all screenings, the tomato varieties SG11, “Moneymaker”, and 142-1007 were used as susceptible controls (Table 1). Healthy seedlings were cultivated and transplanted during the four-leaf growth stage in insect-proof glass greenhouses. By allowing healthy *B. tabaci* biotype *Q* adults to feed on tomato source plants infected with ToCV in an insect-proof cage for 48 h, viruliferous whiteflies were generated. ToCV inoculation was carried out in a glass greenhouse, and infected whiteflies could enter through the open side windows. The experimental design employed a randomized complete block structure, where five clonal replicates per genotype constituted each treatment unit. Triple replication was systematically implemented across all treatment units. The distance between plants within each row measured 0.6 m, while the distance separating adjacent rows was 1.5 m. Starting from the second month following transplantation, the plants in the plots were inspected for symptoms of ToCV at two-week intervals. These experiments were carried out in Langfang, Hebei Province, China, during the autumn of 2016. No insecticides were employed, aiming to create an environment that was favorable for virus infection.

The severity of test plant symptoms was evaluated through a visual scale. A rating of 0 meant that no visible symptoms were present. A score of 1 corresponds to mild symptoms, manifesting as leaf chlorosis and yellowing in the lower leaves of the tomato plants (see Figure S1A). When the score is 2, this indicates moderate symptoms, with chlorosis and yellowing affecting half of the leaves (Figure S1B). A rating of 3 signifies that the entire plant showed chlorosis and yellowing symptoms (Figure S1C). Finally, a score of 4 represents severe symptoms, where the whole plant is chlorotic and yellowed, and the fruits were small and failed to develop properly (Figure S1D). The Disease Severity Index (DSI) was computed according to the formula below: DSI = [Σ (s × n)/(N × S)] × 100, where “s” represents the symptom score; “n” stands for the quantity of plants in the genotypes inoculated with ToCV that have the corresponding symptom scores; “S” is the maximum symptom score, which has a value of 4; and “N” is the overall number of plants of this genotype that were inoculated with ToCV [32]. The DSI is used to gauge the progress of the disease. A DSI of 0 implies that the plants are immune to tomato chlorosis virus (ToCV), denoted as (I). From 0 to 10, it indicates high resistance (HR), 10 to 30 represents resistance (R), 30 to 50 represents moderate resistance (MR), 50 to 70 indicates susceptibility (S), and 70 to 100 means high susceptibility (HS).

### 4.2. Absolute Quantification of ToCV Titer Through qRT-PCR

Total RNA extraction and cDNA synthesis were conducted following the methods previously described by Lu et al. (2021) [33]. In order to construct virus concentration standard curves, the initial step was to synthesize cDNA. This was performed RNA with a quantity of 1 μg, which was extracted from the tomato leaves infected with ToCV at 30 days post-inoculation (dpi). The cDNA served as a template in the process of RT-PCR. In this process, the primers ToCVqF and ToCVqR were utilized, and these primers were designed to target the minor coat protein gene (CPm) of ToCV [34]. Following the manufacturer’s guidelines, the 230 base-pair (bp) amplicon obtained was inserted into the pMDTM18-T vector. Plasmid extraction followed the methods described by Lu et al. (2021) [33]. A Biospec-nano spectrophotometer was employed to quantify the plasmid DNA templates. This template was then utilized to create a virus concentration standard curve. This standard curve was generated using a 10-fold dilution series, with concentrations spanning from 5.6 × 10^3^ to 5.6 × 10^9^ copies/μL. Plasmid copy quantification followed the equation C = (A/B) × 6.02 × 10^23^, where A is the DNA concentration (ng/μL); B is the plasmid molecular weight (g/mol); and C is the molecular copies per μL. For the quantification of viral RNA, primers specific to ToCV (ToCVqF: CTTTCTGGATGGTGTTGCGGC and ToCVqR: TCCCCAACCAATGCGTTT) were utilized. RT-qPCR was performed according to the method previously described by Lu et al. (2021) [33,35]. Negative controls consisted of water and RNA extracted from healthy tomato plants. In every qPCR assay, duplicates were included, and each treatment had five replicates. Samples underwent absolute qPCR to precisely quantify the specific amount of ToCV.

### 4.3. Genetic Mapping of ToCV Resistance Genes and QTL-Seq

LA1028 was hybridized with SG11. The F1 progenies obtained from this cross were then self-pollinated to generate the F2 generation. Likewise, a cross was made between LA0444 and SP15, and the F1 progenies obtained from this cross were then self-pollinated to generate the F2. Subsequently, these F2 populations were utilized for QTL mapping and for conducting bulked segregant analysis (BSA). The ToCV symptom rating scale was used to evaluate the resistance of each plant to ToCV. Extreme trait pools were generated by merging equal quantities of DNA sourced from 15 F2 individuals that exhibited high resistance (characterized by a symptom score of 0) and 15 F2 individuals that were highly susceptible (with a symptom score of 4). The cetyltrimethylammonium bromide (CTAB) method was employed to extract total genomic DNA from the leaves of 235 individuals resulting from the F2 cross SG11 × LA1028 and 199 individuals from the F2 cross SP15 × LA0444 [36]. The concentration, purity, and quality of tomato DNA were examined. A micro-spectrophotometer was utilized to assess the concentration and purity, while 3% agarose gel electrophoresis was carried out to evaluate the quality. Approximately 5–10 µg of DNA, which was extracted from two bulked samples as well as two parental samples, was employed to build pair-end sequencing libraries with a read length of 100 base pairs. These libraries were sequenced individually using the Illumina NovaSeq 6000 platform. The raw data were submitted to the GenBank Short Read Archive under accession number SRP491290.

The clean reads obtained were aligned and quantified against the tomato genome (ITAG4.0) employing Burrows–Wheeler Aligner [37]. SAMTools 1.9 (https://github.com/samtools/samtools, accessed on 1 January 2019) was used to obtain BAM files, and Picard 2.18.21 software (https://github.com/broadinstitute/picard, accessed on 1 January 2019) was used for sorting and comparing to locate duplicate records [38]. To prevent false single-nucleotide polymorphism (SNP) identifications close to insertions and deletions (InDels), re-alignment with the Base Alignment Quality (BAQ) approach was carried out. The Genome Analysis Toolkit (GATK) 4 software (https://gatk.broadinstitute.org/hc/en-us, accessed on 1 January 2019) was utilized for the detection of SNPs and small InDels [39]. The proportion of alleles related to each of the two parental genomes was determined in the following way. First, the number of reads containing a single-nucleotide polymorphism (SNP) when compared to the reference genome sequence (AD_ALT) was counted. Then, this count was divided by the total number of reads (DP). The resulting value, calculated as SNP index = AD_ALT/DP, represented the proportion of these alleles. The SNP index was computed for every position throughout the genome. The SNP index for the entire genome was evaluated using the sliding window approach. By default, the window size was set at 1 Mb, and the step size was 10 kb. Association analysis of SNPs was performed through SNP index algorithms [40]. The formula employed for this analysis was ΔSNP index = SNP index (resistance pool)—SNP index (susceptibility pool). The average values were plotted for each chromosome for identification. We anticipated that for the majority of genomic regions, Δ(SNP index) would equal 0. However, only those regions with high absolute values of Δ(SNP index) would imply a significant contribution to the variation in the trait. We set Δ(SNP index) > 0.5 as the threshold for identifying candidate QTLs.

### 4.4. Molecular Marker Development and PCR

The primers for the InDel markers were developed using Primer 5 software (http://www.premierbiosoft.com, accessed on 1 January 2020) and Primer-BLAST (http://blast.ncbi.nlm.nih.gov/, accessed on 1 January 2020). The polymorphic markers were then used on the genotyping of plants in the F2 population. A linkage analysis was conducted using JoinMap 4.0 [41] to map the ToCV-resistant QTLs in LA0444 and LA1028. InDel markers were first screened for polymorphisms. This screening was performed between SG11 and LA1028, as well as between SP15 and LA0444. Table S1 lists the sequences of these polymorphic markers.

According to the initial mapping outcomes of the F2 population (SG11 × LA1028) acquired via BSA-seq, we developed InDel markers on chromosomes 1 and 11 and constructed a genetic linkage map. Likewise, drawing on the initial mapping results of the F2 population (SP15 × LA0444) derived from BSA-seq, we devised InDel markers on chromosomes 7 and 9 and established a genetic linkage map. QTL analysis was carried out through inclusive composite interval mapping, utilizing the BIP (QTL mapping in the biparental populations) feature of QTL IciMapping 4.1 [42]. The LOD thresholds were computed via 1000 permutation tests (*p* < 0.05) and then employed to identify a potential QTL.

PCR and PCR product purification were performed according to the method previously described by Cao et al. (2019) [43].

### 4.5. RNA Transcriptome Analyses Using RNA-Seq

To investigate the resistance mechanisms and pathogenesis of ToCV, the ToCV—resistant accessions LA0444, LA2157, and LA1028, along with the susceptible accessions S11 and SP15, were chosen. Fifteen and thirty days after ToCV inoculation, total RNA was isolated from the leaves. RNA transcriptome analyses were carried out using three biological replicates. RNA-seq analysis was performed on an Illumina HiSeq platform provided by Biomarker Technologies (Beijing, China). The acquired sequences were submitted to the GenBank Short Read Archive under the accession number SRP491285. RNA-seq analysis and the acquisition of FPKM (fragments per kilobase of transcript sequence per million base pairs sequenced) values were carried out according to the methods previously described by Wei et al. (2024) (Table S2) [44]. The expression of genes was ascertained with the criterion of FPKM > 1.5. Comparison efficiency and sample correlation assessments were employed to select samples suitable for expression profiling analysis (Tables S3–S5). DEGseq was utilized to compute *p*-values through the MA-plot-based method. Fold changes were calculated according to the FPKM values (Table S2). To identify differentially expressed genes (DEGs), thresholds were set as a fold change greater than 2 and a false discovery rate less than 0.01. The Database for Annotation, Visualization, and Integrated Discovery (DAVID) was employed to conduct Gene Ontology (GO) analysis on the DEGs.

## 5. Conclusions

In this study, we conducted screening for ToCV-resistant materials. As a result, we identified two immune accessions (LA2157 and LA0444), which had a DSI value of 0, and five highly resistant accessions (LA1283, LA3858, 162-3, Wo587, and LA1028). These findings provide valuable genetic germplasms for the identification of ToCV resistance genes.

Moreover, a new method was devised to assess the resistance of tomatoes to ToCV. It was noted that tomatoes displayed characteristic pathological symptoms on the 15th and 30th days following ToCV inoculation, which were designated as Stage 1 and Stage 2, respectively. In the susceptible germplasms SG11 and SP15, the ToCV titer in Stage 2 was significantly higher compared to Stage 1. The resistant materials showed low ToCV titers in Stage 1 and Stage 2, which implies that the resistant accessions are capable of keeping the virus levels low. These results suggest that ToCV-resistant materials may achieve resistance by maintaining the viral titer at a low level, which provides new insights into the control of ToCV.

This study identified loci resistant to ToCV by combining QTL-seq with QTL mapping in two F2 populations. These populations were generated from the crosses of SG11 (a susceptible accession) with LA1028 (a resistant accession) and SP15 (a susceptible accession) with LA0444 (a resistant accession). Genetic analysis revealed that the resistance to tomato chlorosis virus (ToCV) in LA1028 and LA0444 exhibited a quantitative nature. This resistance was predominantly controlled by four QTLs: *Qtc1.1* and *Qtc11.1*, which originated from LA1028, and *Qtc7.1* and *Qtc9.1*, which came from LA0444. This provides a theoretical basis for disease-resistant breeding in ToCV.

Subsequently, transcriptome analysis was carried out on two susceptible accessions (SP15 and SG11) and three resistant accessions (LA2157, LA0444, and LA1028). As a result, distinct DEGs and particular biological processes were identified during the two stages of ToCV infection. Our results imply that during Stage 1 of ToCV infection, genes associated with the tomato microtubule system may be upregulated. Additionally, in Stage 2, the genes related to peptidase inhibitors showed lower expression levels in LA1028 and LA2157 compared to the susceptible accessions.

This research offers novel genetic germplasms for ToCV resistance. These resources can be highly useful in ToCV-resistant breeding. The mapping of ToCV resistance QTL provides a theoretical basis and new insights for breeding tomatoes with resistance to ToCV.

## Figures and Tables

**Table 1:** Tomato accession information and disease investigation results.

Accession Name	*Solanum* (Section *Lycopersicon*) Species	DSI	Phenotype
Money maker	*S. lycopersicum*	87.5	HS
NCX3032	*S. lycopersicum*	75	HS
LA0154	*S. lycopersicum*	62.5	S
White Beauty	*S. lycopersicum*	75	HS
031-D52	*S. lycopersicum*	80	HS
Hawaii7998	*S. lycopersicum*	72.5	HS
031-D62	*S. lycopersicum*	25	R
Fla.MH-1	*S. lycopersicum*	62.5	S
LA2823	*S. lycopersicum*	55	S
LA2827	*S. lycopersicum*	45	MR
LA2828	*S. lycopersicum*	75	HS
LA2830	*S. lycopersicum*	40	MR
LA0490	*S. lycopersicum*	25	R
LA2821	*S. lycopersicum*	47.5	MR
LA2830	*S. lycopersicum*	43.8	MR
LA1038	*S. lycopersicum*	84.4	HS
Zaofen2	*S. lycopersicum*	65.6	S
CLN3024F2-104-48-1-0	*S. lycopersicum*	27.5	R
Taotailang+8	*S. lycopersicum*	87.5	HS
SG11	*S. lycopersicum*	90.6	HS
142-1007	*S. lycopersicum*	84.4	HS
162-3	*S. peruvianum*	9.4	HR
LA3858	*S. peruvianum*	4.6	HR
LA0444	*S. peruvianum*	0	I
LA2157	*S. peruvianum*	0	I
Wo015	*S. peruvianum*	37.5	MR
Wo030	*S. peruvianum*	50	S
Wo034	*S. peruvianum*	25	R
Wo587	*S. peruvianum*	3.1	HR
Wo589	*S. peruvianum*	31.3	MR
Wo596	*S. peruvianum*	25	R
Wo606	*S. peruvianum*	37.5	MR
Wo607	*S. peruvianum*	46.9	MR
Wo677	*S. peruvianum*	34.4	MR
Wo688	*S. peruvianum*	40.6	MR
SP15	*S. peruvianum*	80	S
LA1283	*S.corneliomulleri*	4.6	HR
162-5	*S. pimpinellifolium*	69.4	S
LA1589-3	*S. pimpinellifolium*	40	MR
LA0722	*S. pimpinellifolium*	37.5	MR
LA1245	*S. pimpinellifolium*	62.5	S
LA1246	*S. pimpinellifolium*	50	S
LA2093	*S. pimpinellifolium*	68.8	S
LA2097	*S. pimpinellifolium*	50	S
LA0937	*S. pimpinellifolium*	75	HS
LA1969	*S. chilense*	25	R
LA1932	*S. chilense*	25	R
LA1777	*S. habrochaites*	25	R
LA1033	*S. habrochaites*	53.1	S
162-37	*S. habrochaites*	67.9	S
162-38	*S. habrochaites*	45	MR
162-39	*S. habrochaites*	45	MR
162-40	*S. habrochaites*	25	R
162-41	*S. habrochaites*	47.2	MR
162-42	*S. habrochaites*	37.5	MR
LA0317	*S. cheesmanii*	18.8	R
LA1028	*S. chmielewskii*	9.4	HR
LA0716	*S. pennellii*	17.9	R

## Data Availability

The data that support the findings of this study and materials used in this study are available from the corresponding author upon reasonable request. The NGS (Next Generation Sequencing) data from QTL-seq have been uploaded to GenBank Short Read Archive under accession number SRP491290. The RNA-seq data have been uploaded to GenBank Short Read Archive under accession number SRP491285.

## Supplementary Materials

The following supporting information can be downloaded at: https://www.mdpi.com/article/10.3390/ijms26052060/s1.
