# Supplementary material for: Screening for Resistant Germplasms and Quantitative Trait Locus Mapping of Resistance to Tomato Chlorosis Virus
Source: Int J Mol Sci. 2025 Feb 26;26(5):2060. doi: 10.3390/ijms26052060 (PMC11900314; doi:10.3390/ijms26052060)
Supplement: Supplementary file 1 [file ijms-26-02060-s001.zip › Supplementary Figure.pdf]

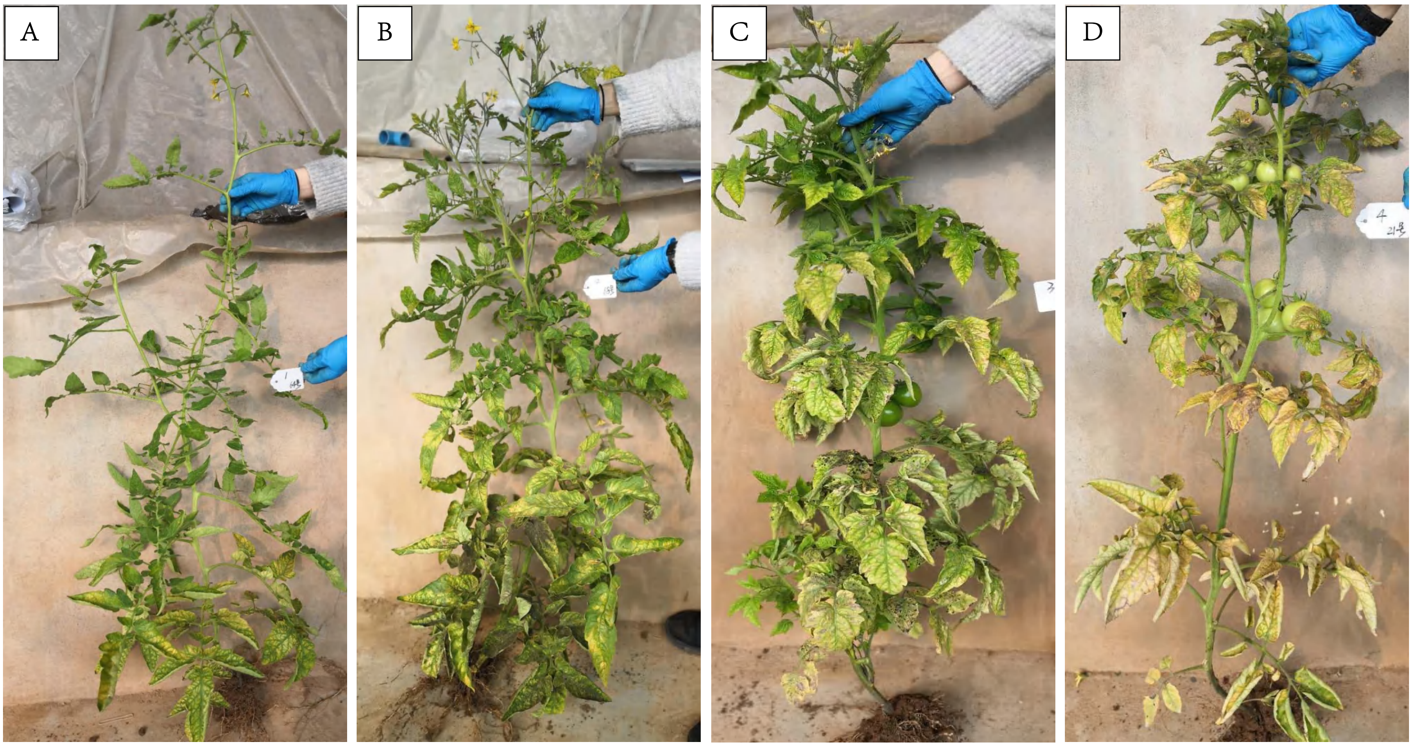

Figure S1 The severity of symptoms in test plants was assessed using a visual scale. (A) symptom score 1, mild symptoms, with the lower leaves of tomato plants exhibiting leaf chlorosis and yellowing. (B) symptom score 2, moderate symptoms, where half of the leaves exhibit chlorosis and yellowing. (C) symptom score 3, chlorosis and yellowing symptoms in the entire plant. (D) symptom score 4 , severe symptoms, with the entire plant displaying chlorosis and yellowing and fruits being small and unable to develop normally

Statistical validation among different ToCV resistance phenotypes (Tukey's Honestly Significant Difference)

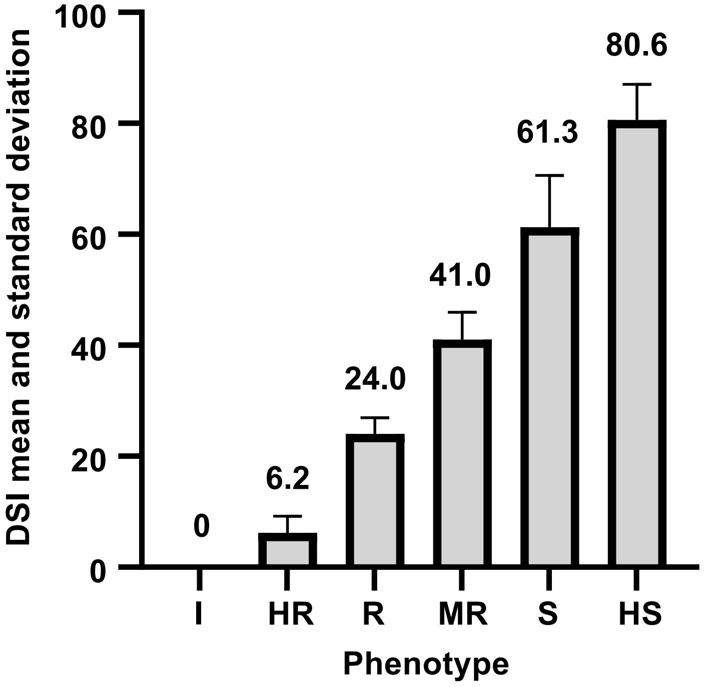

Tukey’s HSD	significant	p-value
I vs HR	ns	0.8239
I vs R	****	<0.0001
I vs MR	****	<0.0001
I vs S	****	<0.0001
I vs HS	****	<0.0001
HR vs R	****	<0.0001
HR vs MR	****	<0.0001
HR vs S	****	<0.0001
HR vs HS	****	<0.0001
R vs MR	****	<0.0001
R vs S	****	<0.0001
R vs HS	****	<0.0001
MR vs S	****	<0.0001
MR vs HS	****	<0.0001
S vs HS	****	<0.0001

Figure S2 Statistical validation among different ToCV resistance phenotypes (Tukey's Honestly Significant Difference)

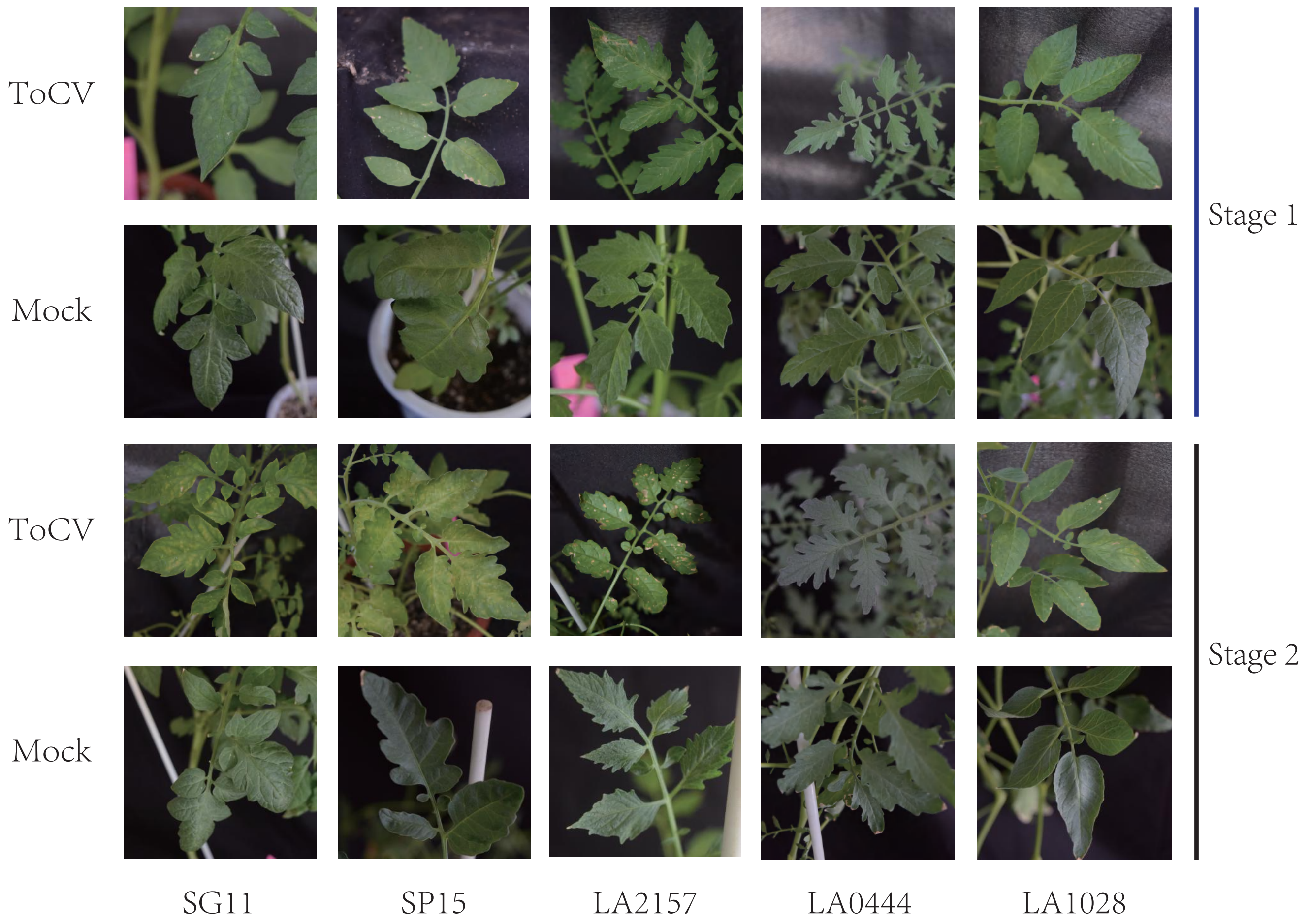

Figure S3 Phenotypes of SG11, SP15, LA2157, LA0444, and LA1028 at stages 1 and 2.

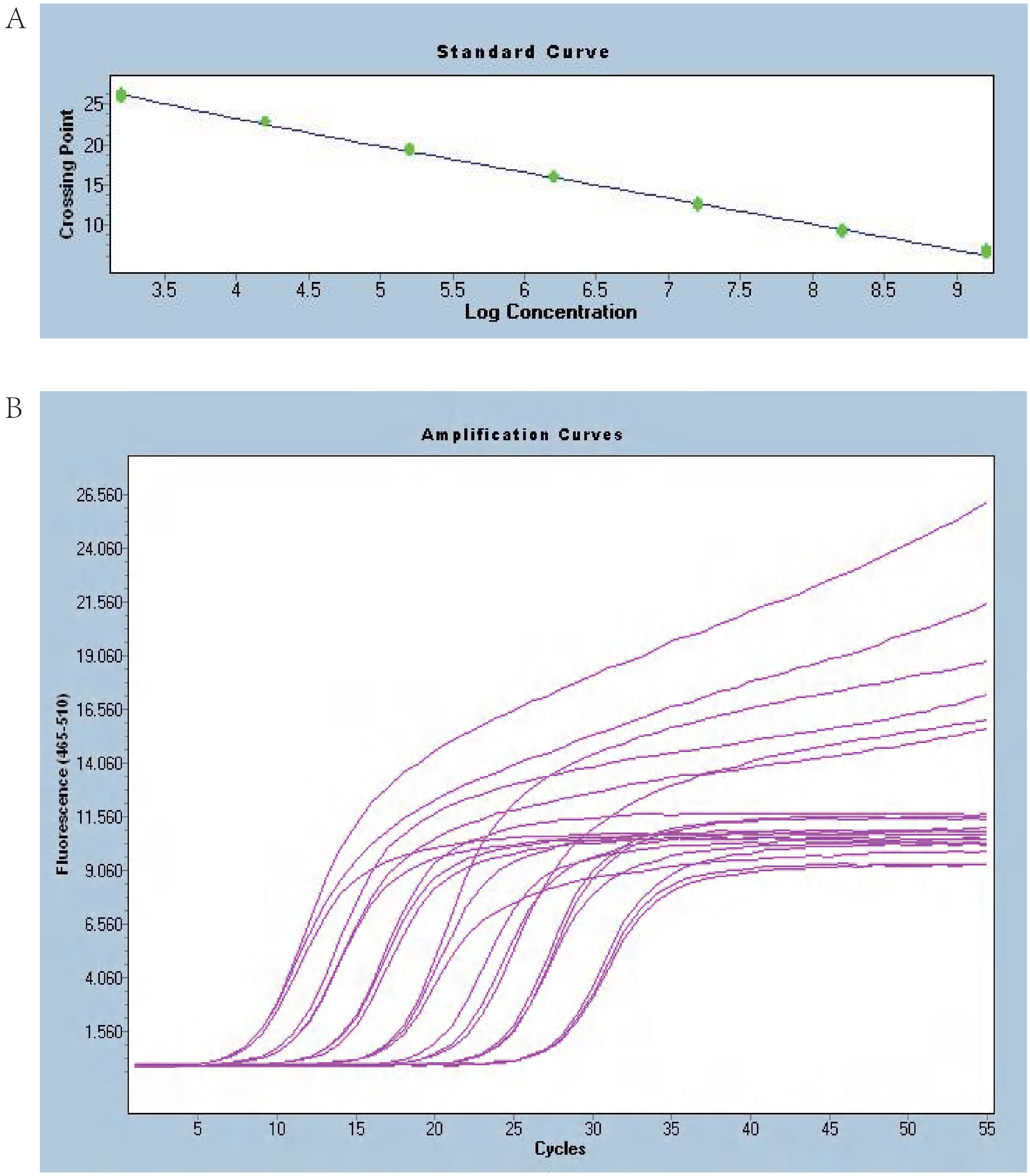

Figure S4 (A) ToCV absolute quantitative standard curve, abscissa is log concentration , ordinate is crossing point. (B) Sensitivity of RT qPCR. The abscissa is the number of cycles, the ordinate is the fluorescence value, and the concentration from right to left is 5.6×103—5.6×109 copies/μL.

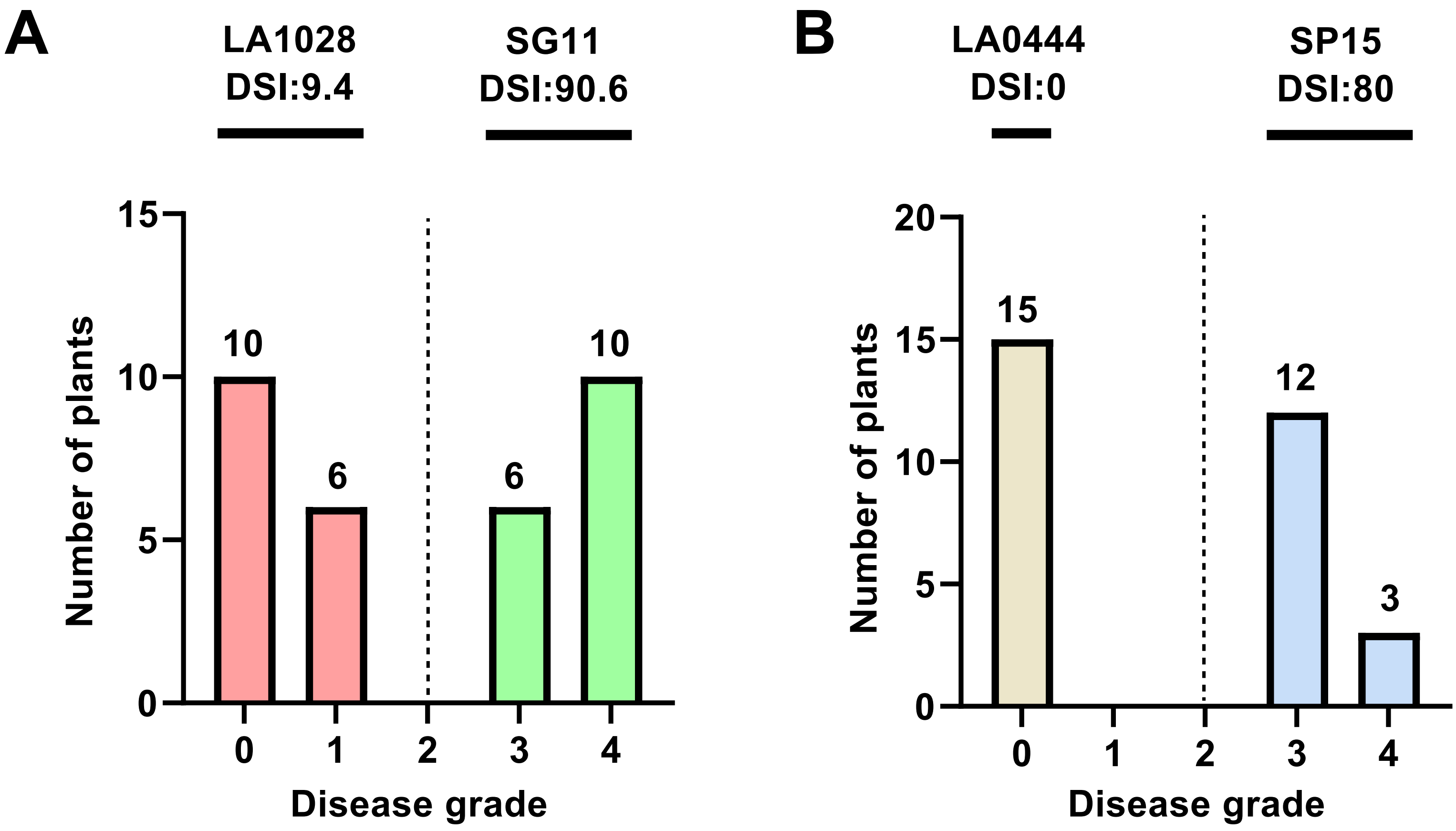

Figure S5 (A) Histogram displaying the distribution of ToCV disease grades of LA1028 and SG11. (B) Histogram displaying the distribution of ToCV disease grades of LA0444 and SP15.

Solyc01g096940

ASPECT	TERM
Cellular Component	**membrane** Source:UniProtKB-KW
Molecular Function	**ATP binding** Source:InterPro
Molecular Function	**protein kinase activity** Source:GO_Central
Biological Process	**defense response to other organism** Source:UniProt

Solyc11g011030

ASPECT	TERM
Cellular Component	**nucleus** Source:GO_Central
Biological Process	**regulation of defense response** Source:GO_Central
Biological Process	**regulation of jasmonic acid mediated signaling pathway** Source:GO_Central
Biological Process	**response to wounding** Source:GO_Central

Solyc09g007190

ASPECT	TERM
Cellular Component	**chloroplast** Source:TreeGrafter

Figure S6 Annotations for Solyc01g096940, Solyc11g011030, and Solyc09g007190.(Data sourced from uniprot: https://www.uniprot.org/)

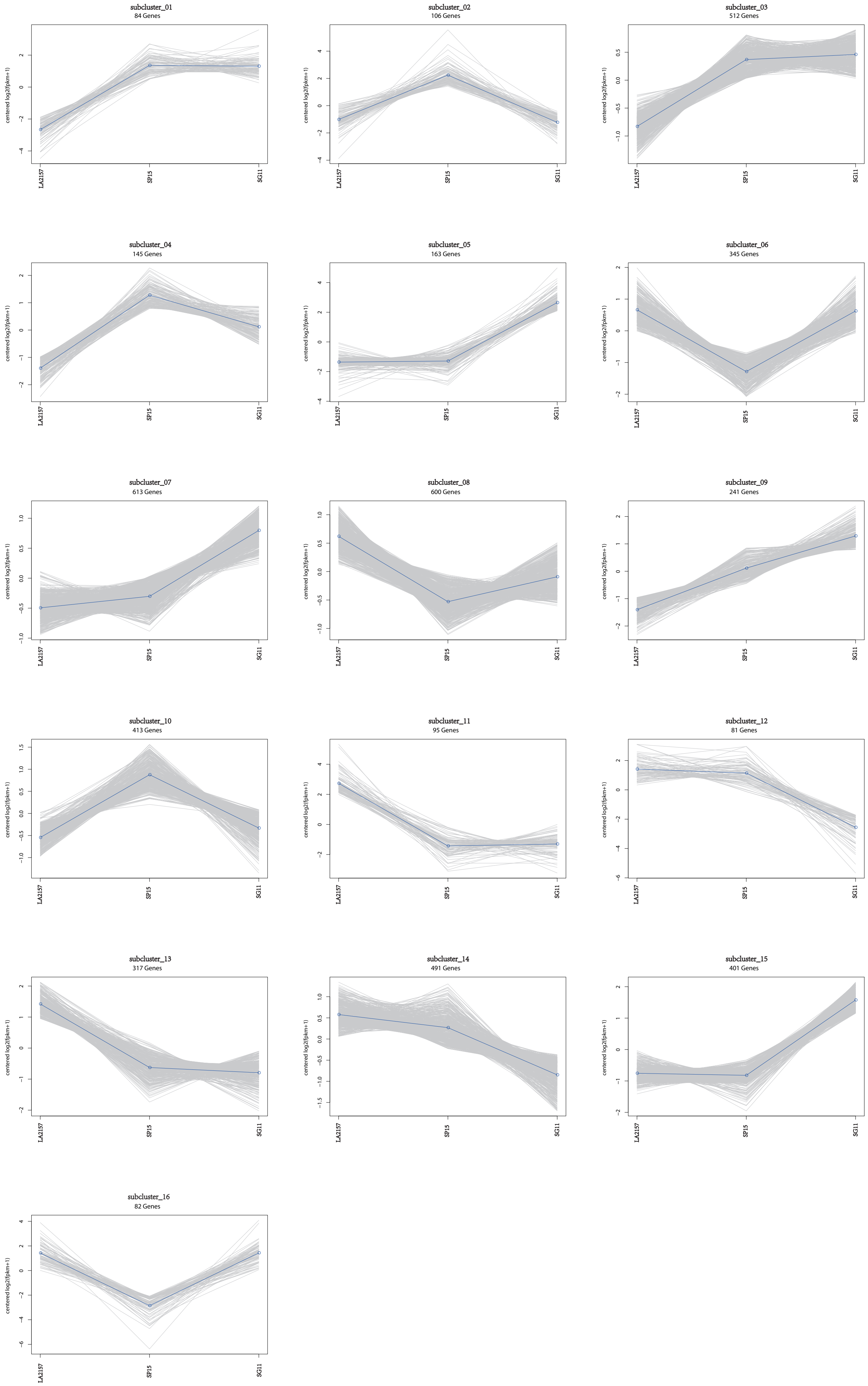

Figure S7 The co-expression trend of LA2157, SG11 and SP15 in stage 1

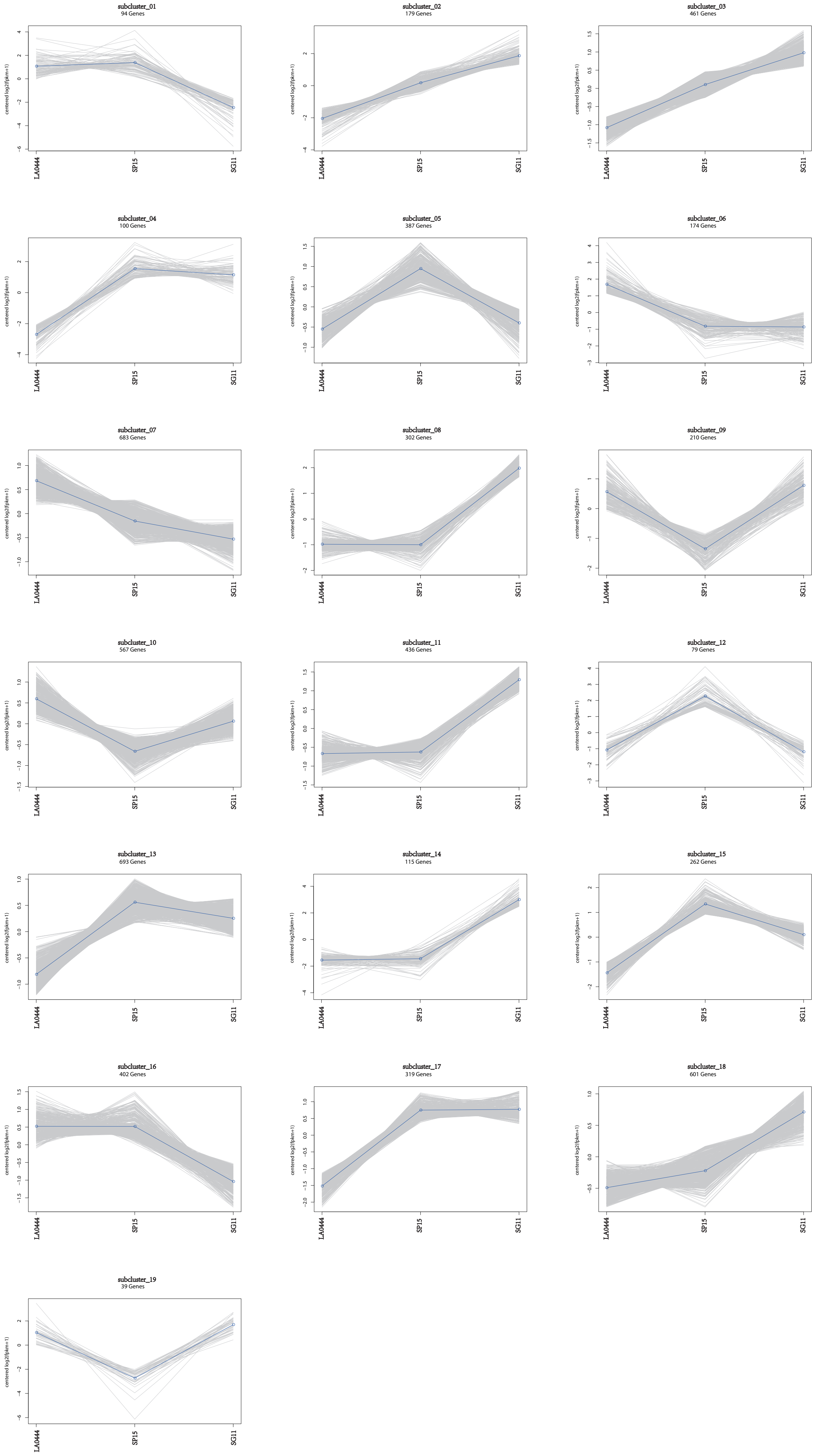

Figure S8 The co-expression trend of LA0444, SG11 and SP15 in stage 1

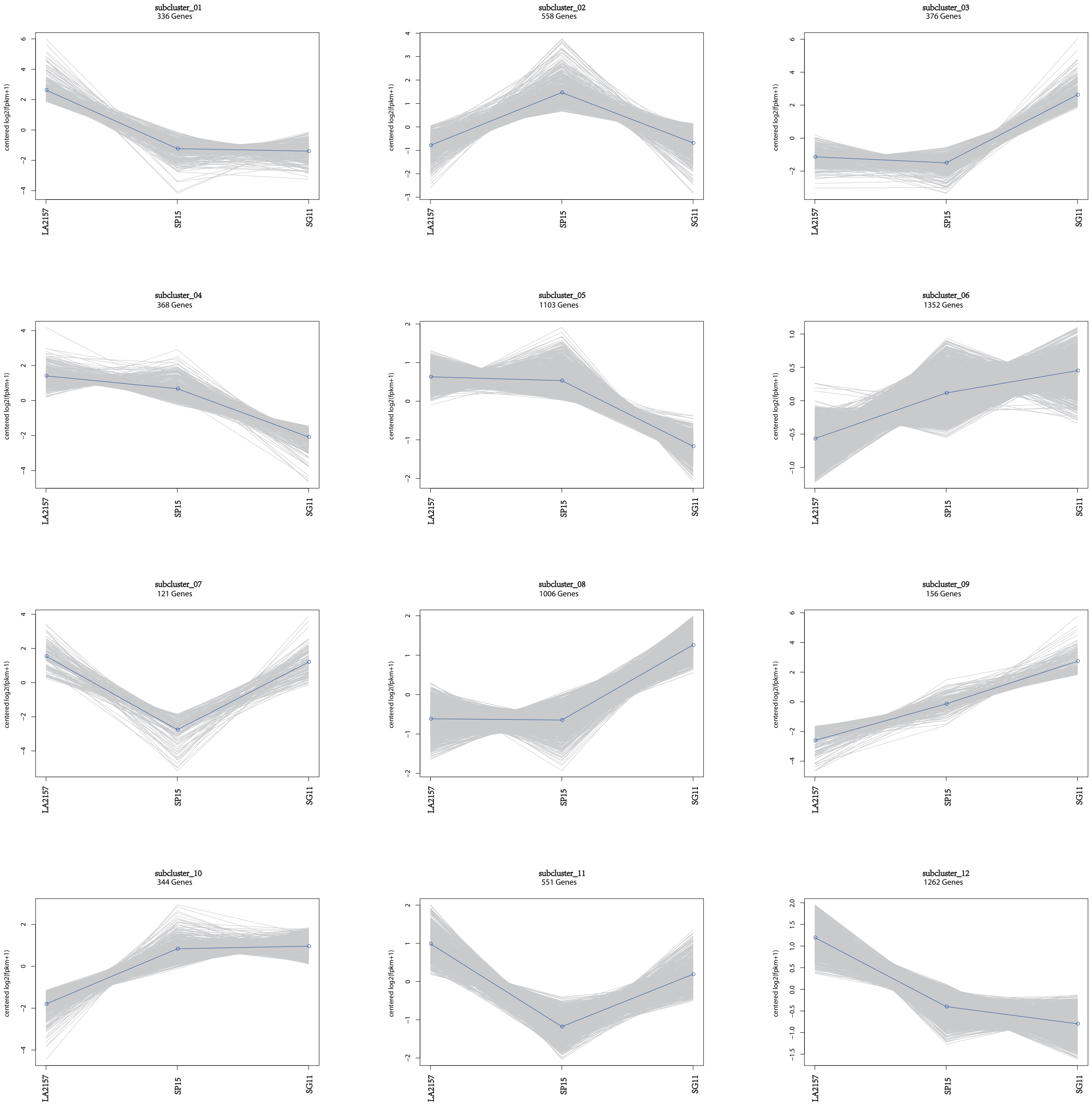

Figure S9 The co-expression trend of LA2157, SG11 and SP15 in stage 2

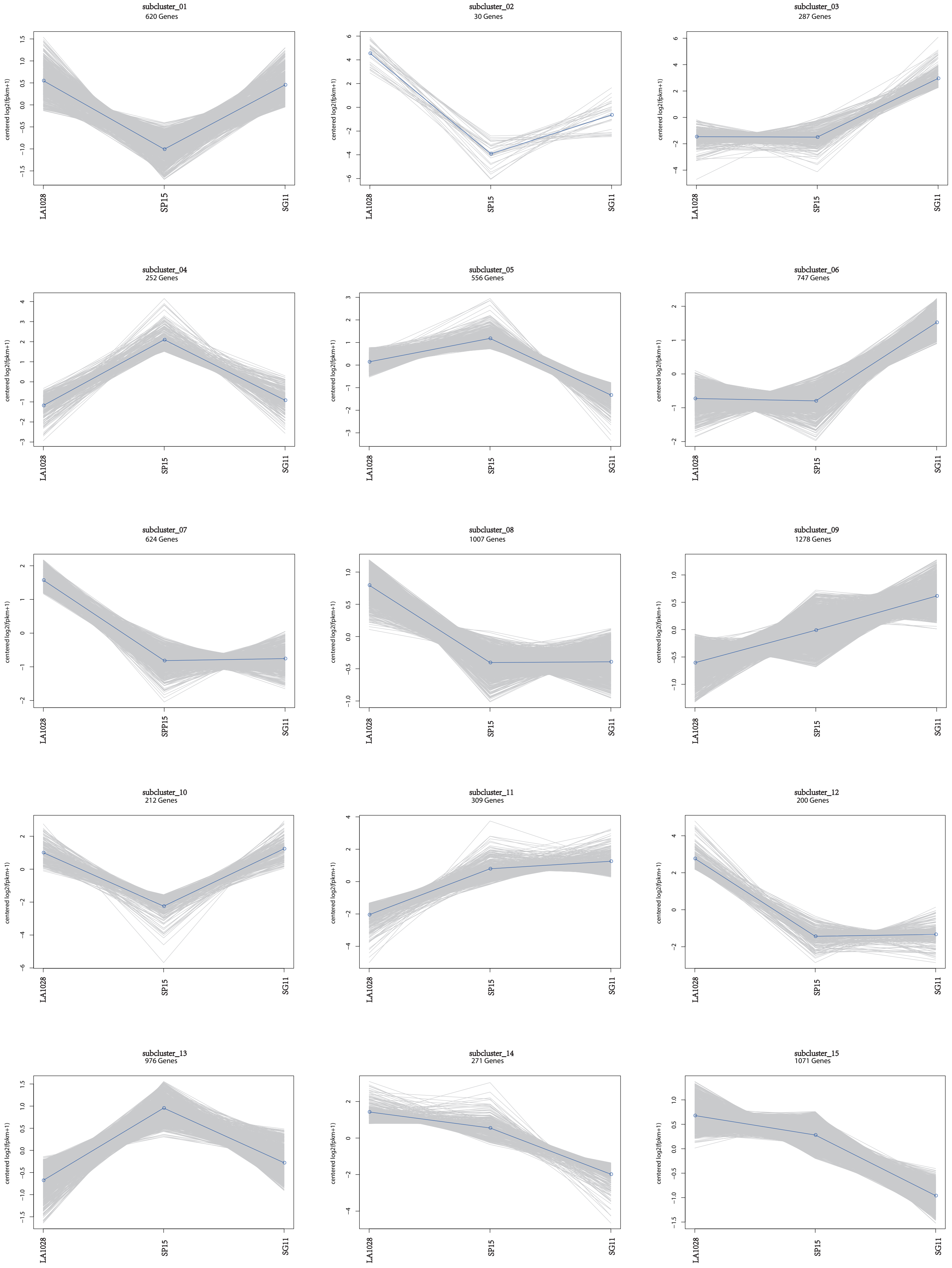

Figure S10 The co-expression trend of LA1028, SG11 and SP15 in stage 2

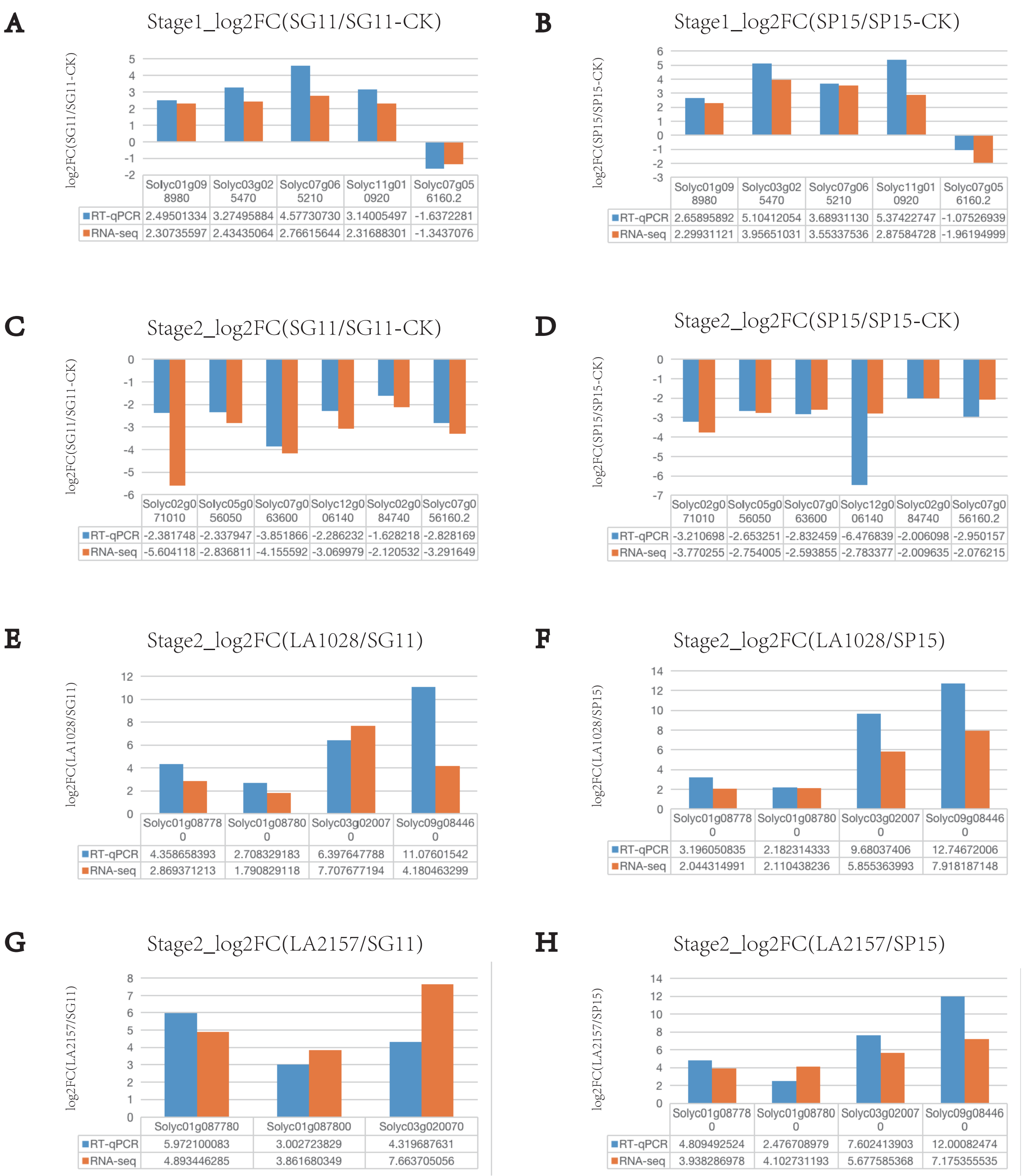

Figure S11 RT-qPCR verification of key genes
